# Clinical utility of the Oncomine Dx Target Test multi‐CDx system and the possibility of utilizing those original sequence data

**DOI:** 10.1002/cam4.7077

**Published:** 2024-03-08

**Authors:** Ayaka Saito, Hideki Terai, Tae‐Jung Kim, Katsura Emoto, Ryutaro Kawano, Kohei Nakamura, Hideyuki Hayashi, Hatsuyo Takaoka, Akihiko Ogata, Katsuhito Kinoshita, Fumimaro Ito, Lisa Shigematsu, Masahiko Okada, Takahiro Fukushima, Akifumi Mitsuishi, Taro Shinozaki, Keiko Ohgino, Shinnosuke Ikemura, Hiroyuki Yasuda, Ichiro Kawada, Kenzo Soejima, Hiroshi Nishihara, Koichi Fukunaga

**Affiliations:** ^1^ Department of Internal Medicine (Pulmonary Medicine), School of Medicine Keio University Tokyo Japan; ^2^ Cancer Center, School of Medicine Keio University Tokyo Japan; ^3^ Department of Hospital Pathology, Yeouido St. Mary Hospital, College of Medicine The Catholic University of Korea Seoul South Korea; ^4^ Division of Diagnostic Pathology, School of Medicine Keio University Tokyo Japan; ^5^ Genomics Unit, Keio Cancer Center, School of Medicine Keio University Tokyo Japan; ^6^ Department of Respiratory Medicine, Graduate School of Medicine University of Yamanashi Yamanashi Japan; ^7^ Keio University Health Center Keio University Tokyo Japan; ^8^ Clinical Translational Research Center Keio University Hospital Tokyo Japan

**Keywords:** *EGFR* exon 19 deletion, lung cancer, ODxTT, PNA‐LNA clamp

## Abstract

**Background:**

Companion diagnostic tests play a crucial role in guiding treatment decisions for patients with non‐small cell lung cancer (NSCLC). The Oncomine Dx Target Test (ODxTT) Multi‐CDx System has emerged as a prominent companion diagnostic method. However, its efficacy in detecting driver gene mutations, particularly rare mutations, warrants investigation.

**Aims:**

This study aimed to assess the performance of the ODxTT in detecting driver gene mutations in NSCLC patients. Specifically, we aimed to evaluate its sensitivity in detecting epidermal growth factor receptor (EGFR) mutations, a key determinant of treatment selection in NSCLC.

**Materials and Methods:**

We conducted a retrospective analysis of NSCLC patients who underwent testing with the ODxTT at Keio University Hospital between May 2020 and March 2022. Patient samples were subjected to both DNA and RNA tests. Driver gene mutation status was assessed, and instances of missed mutations were meticulously examined.

**Results:**

Of the 90 patients, five had nucleic acid quality problems, while 85 underwent both DNA and RNA tests. Driver gene mutations were detected in 56/90 (62.2%) patients. Of the 34 patient specimens, driver mutations were not detected using the ODxTT; however, epidermal growth factor receptor (EGFR) mutations were detected using polymerase chain reaction‐based testing in two patients, and a *KRAS* mutation was detected by careful examination of the sequence data obtained using the ODxTT in one patient. For the above three cases, carefully examining the gene sequence information obtained using the ODxTT could identify driver mutations that were not mentioned in the returned test results. Additionally, we confirmed comparable instances of overlook results for *EGFR* mutations in the dataset from South Korea, implying that this type of oversight could occur in other countries using the ODxTT. *EGFR* mutation was missed in ODxTT in Japan (6.3%, 2/32), South Korea (2.0%, 1/49), and overall (3.7%, 3/81).

**Conclusion:**

Even if sufficient tumor samples are obtained, rare *EGFR* mutations (which are excluded from the ODxTT's genetic mutation list) might not be detected using the current ODxTT system due to the program used for sequence analysis. However, such rare *EGFR* mutations can still be accurately detected on ODxTT's sequence data using next‐generation sequencing.

## Graphical abstract

Pitfalls of Oncomine target testing and the possibility of effective utilization through detailed analysis of original sequencing data.

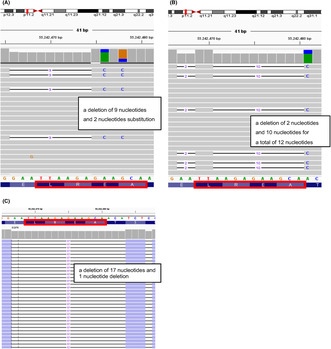

## 1 INTRODUCTION

Lung cancer is the leading cause of cancer‐related death worldwide.^1^ In Japan, more than 120,000 new cases of lung cancer are diagnosed, and 75,000 patients die each year.^2^ Despite recent advances in treating lung cancer, including the development of molecular targeted therapy and immune checkpoint inhibitors, the 5‐year survival rate of patients with advanced lung cancer remains low, and some patients do not respond to any treatment.^1^

Epidermal growth factor receptor (EGFR) mutations are more common in Asians than in Caucasians^3^, ^4^ with a reported frequency of 45% in Japanese patients with adenocarcinoma, the most dominant type of non‐small‐cell lung cancer (NSCLC). EGFR tyrosine kinase inhibitors (TKIs) prolong progression‐free survival and improve the prognosis of patients with NSCLC harboring *EGFR* mutations.^5^, ^6^, ^7^, ^8^ In addition to *EGFR* mutation, other detectable and treatable driver mutations include the *ALK* fusion gene, *ROS1* fusion gene, *BRAF* V600E mutation, *MET* exon 14 skipping mutation, *NTRK* fusion gene, and *RET* fusion gene.^9^

It has been reported that even if patients have driver gene alterations, those who have not used inhibitors of the corresponding driver genes show poor prognosis.^10^ Therefore, identifying driver gene mutations to deliver appropriate targeted treatment is necessary to improve the prognosis of patients with lung cancer. The Oncomine Dx Target Test (ODxTT) multi‐CDx system, a panel‐based gene test using next‐generation sequencing (NGS), was reimbursed for detecting the BRAF V600E by Japanese public insurance in 2018. ODxTT gradually increased the number of genes approved as companion diagnostics; as of February 2023, ODxTT could be used to detect gene alterations of *EGFR*, *ALK*, *ROS1*, *RET*, and *BRAF* simultaneously.^11^, ^12^ Besides being used as a companion diagnostic test, ODxTT can also detect mutations in a total of 46 genes on DNA and RNA. This information about genomic mutations in all 46 genes is returned as reference‐only data for research purposes upon the physician's request.

In Japan, *EGFR* mutations have been mainly detected using polymerase chain reaction (PCR)‐based methods, such as the peptide nucleic acid (PNA)‐locked nucleic acid (LNA) PCR‐clamp method (hereafter referred to as the EGFR clamp method) and other similar methods. These methods are called laboratory‐developed tests (LDTs) and were prevalent until 2016. In 2016, an in vitro diagnostic (IVD) test was launched as a companion diagnostic test for *EGFR* mutations. As a transition period, molecular targeted drugs could be used according to the results of LDTs in clinical practice until March 2022. However, after April 2022, the use of LDTs, including the EGFR clamp method for drug selection as an alternative to companion diagnostic tests, is no longer permitted in Japan.

However, the discrepancy between ODxTT and traditionally used LDTs, such as the EGFR clamp method, has recently been reported.^13^ Driver gene mutations, including *EGFR* mutations, may be positive in LDT tests even though the companion tests failed to detect them. There is growing awareness of the problem of missing driver mutations in companion diagnostic tests.^13^, ^14^ Additionally, with the accumulation of clinical experiences associated with the performance of the ODxTT, unsuccessful testing and obtaining false‐negative and false‐positive results with the ODxTT have been reported for multiple reasons.^13^, ^14^, ^15^

Therefore, in this study, we retrospectively evaluated the performance of the ODxTT. We further examined those samples in which driver gene mutations were not detected on the ODxTT and aimed to clarify the factors that caused the non‐detection of driver gene mutations. Additionally, we compared the results of ODxTT with those of other methods, including the EGFR clamp method. We identified the following two possible causes for missing driver oncogenes using ODxTT:

First, the sequence type of the genomic mutation is not covered by the analysis program in ODxTT. Second, the tumor content was insufficient because of the sparse tumor cells in the tumor. Therefore, if the ODxTT yields a negative result for driver gene mutations, clinicians should determine whether the lung cancer lacks these mutations or whether other factors prevent their detection. We further mention the possibility that even if no driver mutations were detected using ODxTT, the detailed gene sequence information of ODxTT may provide information on the next test that should be performed to detect the driver gene.

## 2 MATERIALS AND METHODS

### 2.1 Patient selection

Between May 2020 and March 2022, 90 patients with NSCLC whose tumor specimens were submitted for ODxTT at Keio University Hospital were enrolled in this study.

### 2.2 Sample specimens

In this study, 90 lung cancer specimens from 90 patients were submitted for the ODxTT at the LSI Medience Corporation. All samples were preserved through formalin fixation and paraffin embedding (FFPE). To ensure the submission of samples with suitable quality and quantity, a pathologist examined the tissue and provided samples containing more than 500 tumor cells and a tumor cell content of over 20%. If the tumor content did not reach 20%, the specific sections for macrodissection were marked by the pathologist. If the specimen was marked, the macrodissection was performed in the LSI Medience Corporation Central Laboratory. Nucleic acid extraction was conducted after the samples were submitted to LSI Medience. Subsequently, the concentration of nucleic acid for the ODxTT was determined using the Ion Torrent Dx DNA and RNA Quantification Kits (A32437 and A32438; Thermo Fisher Scientific, Waltham, USA). If their concentration was lower than the specific threshold (0.83 ng/μl for DNA and 1.43 ng/μl for RNA, which are the minimum required concentrations when using 10 ng of DNA (12 μL) and RNA (8 μL) in the following assay), the test was not conducted.

Additionally, all 90 specimens were sent to the LSI Medience Corporation Central Laboratory for the EGFR clamp assay,^16^, ^17^, ^18^ which was a commonly utilized IVD method for detecting *EGFR* mutations in Japan until March 2022. These assays were conducted between May 2020 and March 2022.

### 2.3 EGFR clamp PCR

The PNA‐LNA PCR clamp is a method for rapid detection of specific mutations or deletions that occur at known positions in the EGFR gene.^16^, ^17^, ^18^ The PNA clamp primer inhibits the amplification of a wild‐type sequence and facilitates the preferential amplification of the mutant sequence. The LNA probe specifically detects the mutant sequence. Since the PNA clamp primer, which carries the wild‐type sequence, and the LNA probe, which carries the mutant sequence, are located at the same position, the PNA clamp primer competitively inhibits the binding of the mutant LNA probe to the wild‐type allele, further enhancing the specificity of detection.

The samples were analyzed in the LSI Medience Corporation Central Laboratory. Data regarding the PCR reaction conditions, primers, and probes are provided in Table S1. The length of PCR amplicons between primers designed for the exon 19 deletion region was assessed using gel electrophoresis to enhance the detection rate of *EGFR* exon 19 deletion. Amplicons with lengths shorter than those of the wild type were classified as mutant variants. The detailed sequence of these mutant amplicons was determined using Sanger sequencing.

### 2.4 Additional data on ODxTT and the PANAMutyper™ EGFR mutation detection test from South Korea

Simultaneous ODxTT and PANAMutyper™ *EGFR* mutation detection tests (Panagene, Daejeon, South Korea) were performed on 218 consecutive samples in Yeouido St. Mary Hospital, South Korea, between December 2021 and December 2022. For sample adequacy, the minimum tumor cell content for proper analysis was determined according to the analytic sensitivity of the testing method.^19^ Tissue input requirement for FFPE sample extraction for ODxTT was determined according to the manufacturer's instructions as follows: 2 × 5 μm sections for resection or surgical biopsies and 9 × 5 μm sections for core needle biopsies. If the tumor content was <20% and the tumor content in the region of interest was ≥10%, the sample was macrodissected and enriched for tumor content, as well as macrodissected for necrotic tissue. Pathologists reviewed hematoxylin and eosin (H&E) stained slides and marked tumor areas directly on the slide. Subsequently, the corresponding areas were manually scraped off from the slides or procured directly from the block using the marked H&E slide as a guide by the technician. The PANAMutyper™ *EGFR* mutation detection test is a PNA‐mediated real‐time PCR method using a PNA clamp and detection probe.^20^, ^21^, ^22^ Regarding sample adequacy for PNA‐mediated real‐time PCR, a minimum of 10% tumor cell content and microdissection was performed on samples with <10% tumor cell content.^23^ Briefly, PCR was performed in a total volume of 25 μL, including template DNA, primers and PNA probe set, and Taqman PCR master mix. The PCR control lacked a PNA probe and contained the wild‐type template. The PCR was performed using the CFX96 PCR detection system (Bio‐Rad, Philadelphia, PA). PCR cycling was conducted under the following conditions: two holding periods of 50°C for 2 min and 95°C for 15 min; 15 cycles of 95°C for 30 s, 70°C for 20 s, and 63°C for 1 min; and 35 cycles of 95°C for 10 s, 53°C for 20 s, and 73°C for 20 s. A melting curve step was prepared (from 35°C to 75°C with temperature increments of 0.5°C for 3 s) to obtain the fluorescence value on all four channels (FAM, ROX, CY5, and HEX). The melting peaks were derived from the melting curve data. Overall, the following two specifically designed PNA oligomers were used in the qPCR: a clamping PNA, which suppresses the amplification of an undesired or wild‐type allele, and a PNA detection probe, which has a fluorophore and quencher group at each terminus of the probe. The mutations detected based on the melting temperature of each tube with fluorescent dye have been reported in a previous study.^24^ The sensitivity of the PANAMutyper™ *EGFR* mutation detection test for detecting the exon 19 deletion and L858R was 0.005%.^24^

### 2.5 Plesission exome test

#### 2.5.1 DNA extraction

Tissue samples collected from patients who underwent surgeries or biopsies were fixed using FFPE. A pathologist evaluated the tumor cell content by examining H&E‐stained slides, which were macro‐dissected to provide >20% tumor cells. Genomic DNA was quantified using Qubit4 Fluorometer (Q33236; Thermo Fisher Scientific) and its quality was assessed based on the DNA integrity number calculated using the Agilent 4150 TapeStation (Agilent Technologies, Santa Clara, United States).

#### 2.5.2 Next‐generation sequencing

Whole‐exome libraries were prepared using the xGen Exome Research Panel version 2 (Integrated DNA Technologies, Inc.) and sequenced using the NovaSeq 6000 system (Illumina) in the 150 bp paired‐end mode. Genome annotation and curation for analyzing the sequencing data were undertaken using an original bioinformatic pipeline created on GenomeJack (Mitsubishi Electric Software Co., Ltd.; http://genomejack.net/english/index.html). In this pipeline, paired reads with a low sequence quality were discarded; subsequently, the NGS reads were mapped to the human reference genome (University of California, Santa Cruz human genome 19) using the Illumina DRAGEN Bio‐IT Platform version 3.8 (Illumina). To identify the single nucleotide variants, SAMtools version 1.9 was used to pile up the sequencing reads, and defective SNVs that showed conflict between pairwise reads were discarded. SAMtools mpileup and VarScan version 2.4.0 mpileup2snp/mpileup2indel were used to identify the variants. The P value obtained using Fisher's exact test in VarScan calling was set to 0.01.

Cancer‐specific somatic gene alterations, including SNVs, indels, and copy number alterations, were identified. All detected gene alterations in 728 census genes (COSMIC version 87) were annotated and curated using the COSMIC (https://cancer.sanger.ac.uk/cosmic), ClinVar (https://www.ncbi.nlm.nih.gov/clinvar/), CIViC (https://civicdb.org/home), SnpEff4.2, and Clinical Knowledgebase (CKB) (https://ckb.jax.org/) databases.

### 2.6 A detailed review of the sequence data of ODxTT using an Integrative Genomics Viewer (IGV)

With the help of the LSI Medience Corporation, we returned the sequence data in the binary version of the sequence alignment data (BAM file). Subsequently, using BAM files of ODxTT, we elucidated the gene sequence using the IGV.^25^

### 2.7 Study oversight

The Ethics Committee of Keio University School of Medicine approved this retrospective observational study (approval numbers: 2022‐1028 and 2011‐0171), and the requirement for obtaining written informed consent was waived. Furthermore, we collected clinical information from eligible individuals with the opportunity for patients to refuse to participate in the study (opt‐out method). Sequence data were obtained from Yeouido St. Mary Hospital. This study was also approved by the Institutional Review Board of Yeouido St. Mary Hospital (approval number: SC18RNSI0037).

### 2.8 Statistical analysis

The chi‐square test was used to analyze the success rates. Statistical significance was set at *p* < 0.05. All statistical analyses were performed using Prism version 9 (GraphPad Software, Boston, MA, USA).

## 3 RESULTS

### 3.1 Baseline characteristics

The background characteristics of the 90 patients whose samples were subjected to ODxTT are presented in Table 1. The patients' median age was 69 years (range: 27–89 years), 52 (57.8%) were male, 63 (70.0%) were smokers, and 65 (72.2%) had the most dominant histological type of lung adenocarcinoma.

**TABLE 1:** Clinical background of patients who underwent ODxTT testing.

Keio University Hospital in Japan	
Characteristics	Number (%)
Age	
Median (range)	69 (27–89)
Sex	
Male	52 (57.8)
Female	38 (42.2)
Smoking status	
Never smoker	27 (30.0)
Ex‐ or current‐smoker	63 (70.0)
Histology	
Adenocarcinoma	65 (72.2)
Squamous cell carcinoma	14 (15.6)
Not otherwise specified	4 (4.4)
Others	7 (7.8)

Yeouido St. Mary Hospital in South Korea	
Characteristics	Number (%)
Age	
Median (range)	67 (38–87)
Sex	
Male	129 (59.2)
Female	89 (40.9)
Smoking status	
Never smoker	102 (46.8)
Ex‐ or current‐smoker	116 (53.2)
Histology	
Adenocarcinoma	162 (74.3)
Squamous cell carcinoma	42 (19.3)
Not otherwise specified	0 (0.0)
Others	14 (6.4)

Abbreviation: ODxTT, Oncomine Dx Target Test.

### 3.2 Specimen type and success rate of ODxTT

Among the 90 specimens from 90 patients, only 85 (94.4%) were successfully analyzed for both DNA and RNA; relevant mutations were not detected by measuring the RNA, DNA, and both DNA and RNA expressions in three (3.3%), one (1.1%), and one (1.1%) samples, respectively.

**FIGURE 1:**
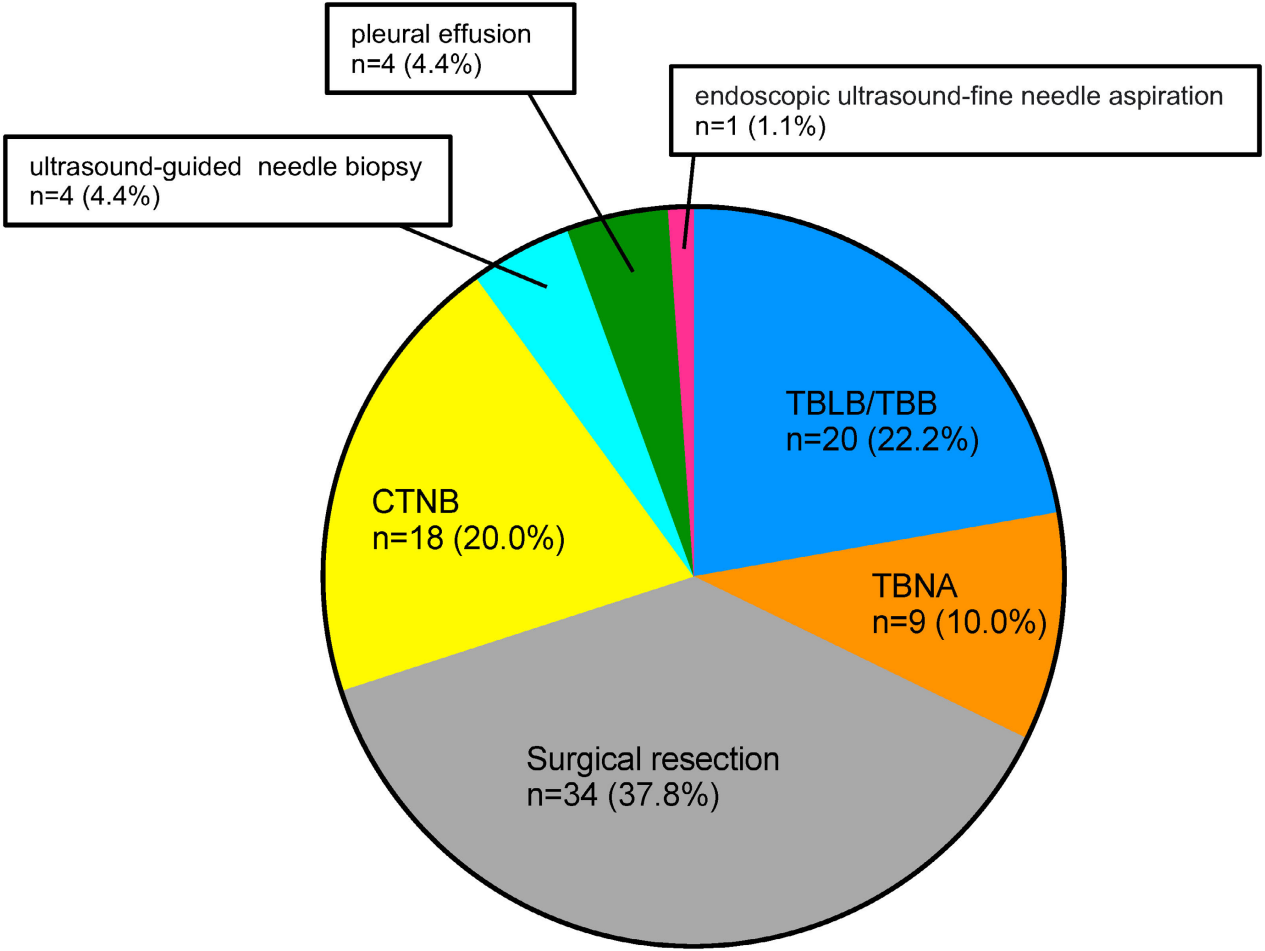
Distribution of procedures to obtain each specimen. CTNB, computed tomography (CT)‐guided needle biopsy; EBUS‐TBNA, endobronchial ultrasound‐guided, transbronchial needle aspiration; TBB, transbronchial biopsy; TBLB, transbronchial lung biopsy.

The procedures performed to obtain each specimen were as follows: transbronchial lung biopsy, ultrasound bronchoscopy‐guided biopsy, surgical resection, computed tomography (CT)‐guided biopsy, ultrasound‐guided biopsy, pleural fluid cell block, and endoscopic ultrasound‐guided needle biopsy in 20 (22.2%), 9 (10.0%), 34 (37.8%), 18 (20.0%), 4 (4.4%), 4 (4.4%), and 1 (1.1%) cases, respectively (Figure 1).

At our institution, the success rate of the ODxTT in the samples obtained through surgical resection (29/34 patients) was relatively low. However, samples obtained through bronchoscopy, CT‐guided needle biopsy, or other procedures were successfully analyzed (56/56 cases) (Table S2). Detailed information on the failure cases is provided in Table S3. The minimum amount of DNA required for detecting *EGFR* mutations in ODxTT was 10 ng.^26^ As mentioned in the Materials and Methods section, at least 0.83 ng/μL of DNA and 1.43 ng/μL of RNA were required for testing. Even after submitting sufficient amounts of nucleic acid samples to LSI Medience, five specimens still failed to show positive results on ODxTT. However, since the quality of the nucleic acid has not been evaluated, we cannot rule out the potential influence of its quality on the test result.

### 3.3 List of driver mutations detected using ODxTT

**FIGURE 2:**
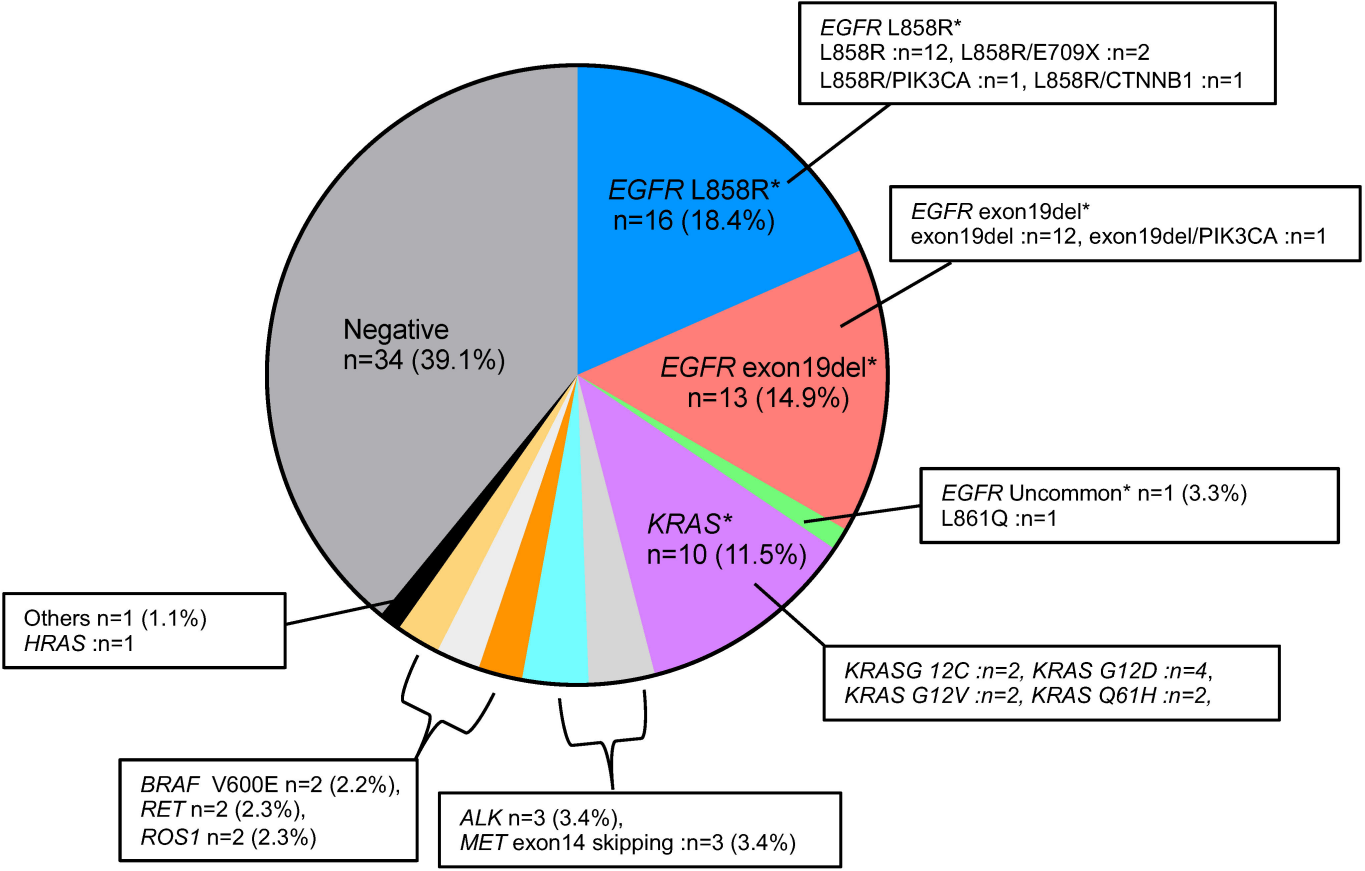
Distribution of driver mutations detected using ODxTT. The distribution of driver gene mutations detected using ODxTT at Keio University Hospital is shown in a pie chart (*n* = 90). ODxTT, Oncomine Dx Target Test.

Driver mutations were confirmed in 53 (58.9%) patients. *EGFR* mutations were detected in 30 (33.3%) patients. Other detected driver mutations were as follows: *KRAS* mutations, *BRAF* mutations, *ALK* fusion genes, *RET* fusion genes, and *MET* exon 14 skipping mutations in 10 (11.1%), 2 (2.2%), 3 (3.3%), 2 (2.2%), and 3 (3.3%) patients, respectively. However, in the remaining 34 (37.8%) patients, no driver mutations were detected on ODxTT (Figure 2).

### 3.4 Discrepancies in detecting the frequency of EGFR mutations between ODxTT and EGFR clamp method

We focused on the discrepancies between the ODxTT and PCR tests. First, we focused on the samples analyzed using both ODxTT and EGFR clamp tests at Keio University. *EGFR* mutations were detected in 30/90 (33.3%) and 32/90 (35.6%) patients using ODxTT and the EGFR clamp method, respectively (*p* = 0.754, chi‐square test). Although no significant difference was found in the number of samples examined using the ODxTT and PCR methods, ODxTT failed to detect *EGFR* mutations in two samples (both had *EGFR* Exon 19 deletions). Accordingly, compared with the EGFR clamp test, the detection rate of *EGFR* mutations with ODxTT was 93.9%. The proportion of *EGFR* mutations detected using the ODxTT and EGFR clamp method is presented in Table 2.

**TABLE 2:** Detailed information on patients' data whose *EGFR* driver mutation was not detected using the ODxTT.

		ODxTT	
		Positive (*n* = 30)	Negative (*n* = 60)
EGFR clamp	Positive (*n* = 32)	30	2
	Negative (*n* = 58)	0	58

Abbreviations: EGFR, epidermal growth factor receptor; ODxTT, Oncomine Dx Target Test.

### 3.5 Additional comparison data of ODxTT and PCR‐based detection method from South Korea

ODxTT may fail to detect specific types of *EGFR* mutations, although these mutations were detected using the PCR‐based detection method. We speculated whether this issue could be generalized to other countries. Therefore, we obtained data from Yeouido St. Mary Hospital in South Korea. Consistent with our findings, ODxTT also failed to detect *EGFR* mutations in this dataset, although the PCR‐based detection method PANAMutyper successfully detected the mutation in the same samples. In 218 samples analyzed using both ODxTT and PANA Mutyper, *EGFR* mutations were detected in 49 samples, and ODxTT failed to detect one *EGFR* mutation compared with PANAMutyper (*p* > 0.999, chi‐square test) (Table S4). Similar to the comparison with the EGFR clamp method, when compared to the PANAMyer method, the ODxTT test was compatible with detecting the *EGFR* driver gene. However, *EGFR* mutations were not detected in some patients during ODxTT, although such cases were limited.

### 3.6 Treatment effects in patients with EGFR mutation, which were not detected using ODxTT

We also evaluated the treatment effects of EGFR TKIs in patients with these *EGFR* mutations (Table 3). In cases #1, #2, and #3, osimertinib was used as first‐line therapy. The best response was a partial response (PR) in all three cases. Since the anti‐tumor effect of osimertinib was clearly observed, it was concluded that the *EGFR* mutations detected using the PCR‐based method were functional.

**TABLE 3:** Detailed information on the cases in which ODxTT failed to detect *EGFR* mutations.

No	Age	Sex	Smoking status	Histology	ODxTT	PCR detection method	*EGFR* mutation status	Gene	EGFR‐TKI	Line of therapy	Response	Duration of TKI therapy (months)
1	53	M	Smoker	Adenocarcinoma	Not detected	EGFR Clamp	p.E746_A750delinsAP	Exon19 del	Osimertinib	1	PR	8.0
2	73	M	Non‐smoker	Adenocarcinoma	Not detected	EGFR Clamp	p.E746_A751delinsVP	Exon19 del	Osimertinib	1	PR	Not reached
3	68	F	Non‐smoker	Adenocarcinoma	Not detected	PANAMutyper	p.E746_A753delinsVS	Exon19 del	Osimertinib	1	PR	6.0

Abbreviations: EGFR, epidermal growth factor receptor; ODxTT, Oncomine Dx Target Test.

### 3.7 Confirmation of sequence results through an IGV viewer in each case

We obtained the BAM files of the sequencing data to elucidate the sequence results of three overlooked cases using ODxTT. With careful observation of BAM files using the sequence viewer IGV, we successfully visualized *EGFR* exon 19 deletion in all three cases. The detailed types of *EGFR* mutations that were not detected using ODxTT are summarized in Table 3. These rare *EGFR* exon 19 deletions were not included in this report because they were excluded from the list of mutations targeted by ODxTT.

**FIGURE 3:**
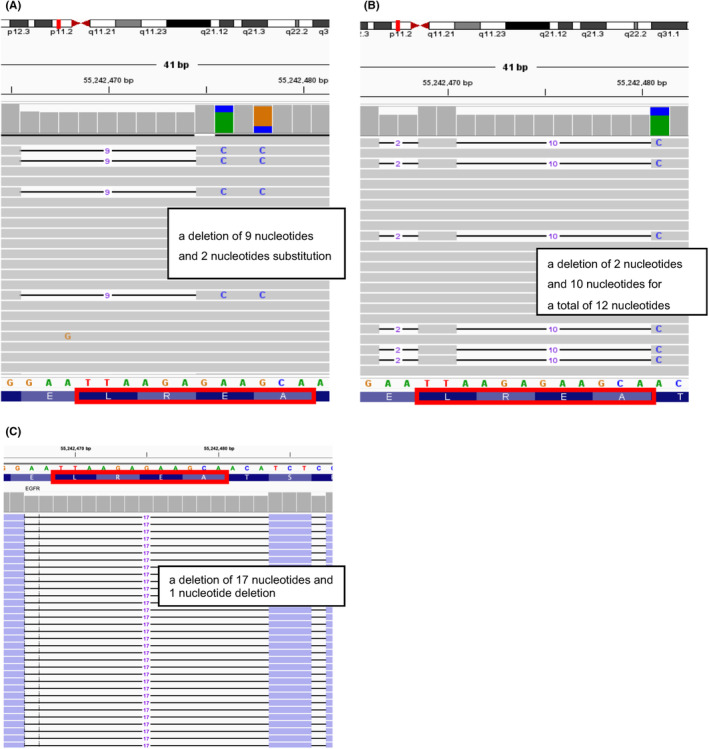
Detailed sequence information of the cases in which ODxTT failed to detect *EGFR* exon 19 deletions. Sequencing information visualized using an integrated genome viewer of cases #1 (A), #2 (B), and #3 (C) is shown. ODxTT, Oncomine Dx Target Test; EGFR, epidermal growth factor receptor.

In patient #1, a rare type of *EGFR* mutation [E746_A750 delins AP mutations] was detected (Figure 3A).

In patient #2, a rare type of *EGFR* mutation [E746_A751 delins VP mutations] was detected. Therefore, a small double deletion (a deletion of 2 and 10 nucleotides for a total of 12 nucleotides) occurred in exon 19 (Figure 3B).

In patient #3, a rare type of *EGFR* mutation [E746_A753 delins VS mutations] was detected. This mutation was also a double small deletion (a deletion of 17 and 1 nucleotide for a total of 18 nucleotides) in exon 19 (Figure 3C).

These three types of mutations cannot be detected using ODxTT^27^ (Table S5).

### 3.8 Additional case with potential driver oncogene detected by IGV viewer

In case #4 with invasive mucinous adenocarcinoma, ODxTT possibly did not detect driver mutations, although this type of lung cancer mostly harbors *KRAS* mutations.^28^ Therefore, to investigate unknown driver mutation, the same sample was submitted after microdissection for whole exome‐sequencing using the Plesission Exome test, which was developed at Keio University Hospital.^29^ The H&E‐stained images before and after macrodissection are shown in Figures S1 and S2, respectively. The tumor content in specimens increased from 10% to 20% following microdissection (Figures S1 and S2).

Therefore, the *KRAS* G12V mutation was confirmed using Plesission Exome sequencing with a coverage of 346 at the KRAS p.G12V site and a variant allele frequency of 9.5% (33/346) (Figure S3A; Table S6). Although specific numerical values for determining gene mutation detection on ODxTT have not been publicly disclosed, multiple factors, including the sequence depth in the relevant region, mutation allele frequency, and the balance between reads on the plus (+) and minus (−) strands would influence the decision.

With the analysis of ODxTT sequence data in this case, the sequencing depth in the relevant region was sufficient; however, the mutation allele frequency was only 3%, which was relatively low (Figure S3B). In retrospect, macrodissection could be warranted when submitting this case for the ODxTT analysis. ODxTT could detect *KRAS* G12V in this case with increased tumor fraction by microdissection.

## 4 DISCUSSION

In this study, we assessed the feasibility of ODxTT in a real‐world clinical setting. Because we submitted specimens selected by a pathologist to judge whether they contained sufficient tumor cell content, most of the samples were sequenced appropriately. However, several specimens could not achieve a sufficient quality or quantity of nucleotide reads for the ODxTT. All samples that failed to undergo the ODxTT test due to unsatisfactory nucleic acids were surgical specimens at our institution. Furthermore, we observed two cases where we failed to detect driver mutations using ODxTT, although the EGFR clamp method successfully detected *EGFR* mutations in the same samples. Additionally, with data from South Korea, ODxTT failed to detect *EGFR* mutations, although the PCR‐based method PANAMutyper successfully detected these mutations in the same sample. These data suggest a difference in the ability of ODxTT and PCR‐based detection methods to detect driver mutations.

We evaluated similar overlooked cases by ODxTT from the literature, which are summarized in Table 4. Consistent with our findings, several previous reports have also indicated that ODxTT may not be comparable to conventional single‐gene testing for detecting *EGFR* mutations.^13^, ^14^ For example, Sakaguchi et al. reported that among 116 patients with *EGFR* mutations detected using the EGFR clamp test, ODxTT failed to detect seven (6%) *EGFR* mutations.^13^ Additionally, Murakami et al. reported that among 200 patients with *EGFR* mutations detected using Cobas, ODxTT failed to detect two (1%) *EGFR* mutations.^14^

**TABLE 4:** Comparison of false negative rates of ODxTT, testing methods, and mutations in studies reported to date.

References	Disconcordance		Method	Mutation status	Potential cause of disconcordance
	Percentage in total sample	Percentage of *EGFR* mutation			
Ariyasu et al.^12^	–	0% (0/25)	ODxTT versus cobas® EGFR	–	
Sakaguchi et al.^13^	6% (7/116)	–	ODxTT versus EGFR clamp	Exon 19 del	Low sensitivity of ODxTT
				Exon 19 del	Low sensitivity of ODxTT
				Exon 19 del	Cause unknown
				Exon 19 del	Cause unknown
				Exon 20 ins	Out of the detection coverage by ODxTT
Murakami et al.^14^	1.0% (2/200)	5.4% (3/56)	ODxTT versus cobas® EGFR	Exon 19 del	Out of the detection coverage by ODxTT
				Exon 19 del	Out of the detection coverage by ODxTT
Present study (Keio University Hospital)	2.2% (2/90)	6.1% (2/33)	ODxTT versus EGFR clamp	Exon 19 del	Out of the detection coverage by ODxTT
				Exon 19 del	Out of the detection coverage by ODxTT
Present study (The Catholic University of Korea)	0.5% (1/218)	2.0% (1/49)	ODxTT versus PANA Mutyper	Exon 19 del	Out of the detection coverage by ODxTT

Abbreviation: ODxTT, Oncomine Dx Target Test.

Since previous studies did not mention the mechanism by which ODxTT failed to detect these rare variants of mutations,^13^, ^14^ we performed a detailed analysis of the original sequencing data of ODxTT. The different types of *EGFR* exon 19 deletions detected using ODxTT, cobas EGFR version 2, therascreen EGFR RGQ, and Amoy Dx Pan Lung Cancer PCR Panel are summarized in a previous report.^27^ None of these companion diagnostic tests covered the types of *EGFR* exon 19 deletions detected in cases #1–#3. In contrast, the EGFR clamp method successfully detected these variants, suggesting that the EGFR clamp method has better detection capabilities than these companion diagnostic tests to detect some of the rare variants of *EGFR*. Since the EGFR clamp method is a highly sensitive detection method based on wild‐type allele‐specific PCR inhibition (not mutant‐type specific detection method),^16^, ^30^ various mutation types could be detected using the PCR clamp method. We also confirmed from LSI Medience (the supplier of the EGFR clamp method kit) that the EGFR clamp method could detect these rare types of mutations (Table S4).

In this study, we identified two instances where ODxTT failed to detect driver mutations. First, the sequencing analysis program failed to detect rare mutation variants, although it was sequenced appropriately with the sample. Second, ODxTT failed to detect a few cancer cells in the tumor tissue.

However, clinicians could not determine whether the test failed due to the insufficient tumor content of the sample or an inadequately developed analysis method. Therefore, raw or detailed sequencing data for ODxTT would be helpful in determining the appropriate method to detect driver oncogenes in these cases.

Recently, the therapeutic approach for patients with advanced lung adenocarcinoma has markedly changed with the use of molecular‐targeted drugs. The median survival time was extended to 3.5 years in patients whose driver mutations were detected, and appropriate targeted therapy was provided. In contrast, the median survival time was 2.5 years in cases where driver mutations were detected; however, no targeted therapy was administered. This survival time is close to the median survival time of 2.1 years in cases where no driver mutations were detected.^11^

Overlooked results may deprive patients of the opportunity to receive targeted therapy, highlighting the importance of conducting additional tests if the ODxTT test is negative. On the other hand, it might be possible to identify these overlooked driver gene mutations using alternative methods, including PCR; however, additional testing would incur extra costs. The sequencing data of ODxTT would be helpful to speculate about potential driver oncogenes without incurring significant additional costs or requiring additional specimens, even if these sequences of mutations were not approved as companion diagnostic tests. Furthermore, considering that genetic mutations can be confirmed using IGV, improving the programs for analyzing the sequence results, such as continuous updating and registration of rare genetic mutations, could lead to more efficient detection of driver gene mutations from the ODxTT sequence data.

This study had some limitations. The study is based on data from two centers in two countries and not all cases were tested using ODxTT in parallel with PCR‐based testing. More detailed sequencing analysis may be necessary in clinical settings where comprehensive genomic analysis has been implemented. Therefore, with these limitations, further research will be necessary to draw more clinically relevant conclusions.

In conclusion, ODxTT could detect driver gene mutations in 58.5% of cases, whereas there were some cases of *EGFR* mutations where the results of the ODxTT and EGFR clamp assays diverged, all of which were exon 19 deletions. Therefore, it is crucial to perform additional tests, such as oncogene panel testing, in patients suspected to be *EGFR* mutation‐positive, even if the ODxTT test is negative.

## AUTHOR CONTRIBUTIONS

**Ayaka Saito:** Data curation (lead); formal analysis (lead); investigation (lead); methodology (lead); project administration (lead); writing – original draft (lead); writing – review and editing (lead). **Hideki Terai:** Conceptualization (lead); data curation (lead); formal analysis (lead); investigation (lead); methodology (lead); project administration (lead); writing – original draft (lead); writing – review and editing (lead). **Tae‐Jung Kim:** Conceptualization (equal); data curation (equal); investigation (equal); methodology (equal); writing – review and editing (equal). **Katsura Emoto:** Data curation (equal); investigation (equal); methodology (equal); writing – review and editing (equal). **Ryutaro Kawano:** Data curation (equal); investigation (equal); methodology (equal); writing – review and editing (equal). **Kohei Nakamura:** Data curation (equal); investigation (equal); methodology (equal); writing – review and editing (equal). **Hideyuki Hayashi:** Conceptualization (equal); data curation (equal); investigation (equal); methodology (equal); writing – review and editing (equal). **Hatsuyo Takaoka:** Data curation (equal); investigation (equal); methodology (equal); writing – review and editing (equal). **Akihiko Ogata:** Data curation (equal); investigation (equal); methodology (equal); writing – review and editing (equal). **Katsuhito Kinoshita:** Data curation (equal); investigation (equal); methodology (equal); writing – review and editing (equal). **Fumimaro Ito:** Data curation (equal); investigation (equal); methodology (equal); writing – review and editing (equal). **Lisa Shigematsu:** Data curation (equal); investigation (equal); methodology (equal); writing – review and editing (equal). **Masahiko Okada:** Data curation (equal); investigation (equal); methodology (equal); writing – review and editing (equal). **Takahiro Fukushima:** Data curation (equal); investigation (equal); methodology (equal); writing – review and editing (equal). **Akifumi Mitsuishi:** Data curation (equal); investigation (equal); methodology (equal); writing – review and editing (equal). **Taro Shinozaki:** Data curation (equal); investigation (equal); methodology (equal); writing – review and editing (equal). **Keiko Ohgino:** Formal analysis (equal); methodology (equal); writing – review and editing (equal). **Shinnosuke Ikemura:** Conceptualization (equal); data curation (equal); investigation (equal); methodology (equal); writing – review and editing (equal). **Hiroyuki Yasuda:** Conceptualization (equal); data curation (equal); funding acquisition (equal); investigation (equal); methodology (equal); project administration (equal); writing – original draft (equal); writing – review and editing (equal). **Ichiro Kawada:** Data curation (equal); investigation (equal); methodology (equal); writing – review and editing (equal). **Kenzo Soejima:** Data curation (equal); investigation (equal); writing – review and editing (equal). **Hiroshi Nishihara:** Conceptualization (equal); data curation (equal); investigation (equal); methodology (equal); writing – review and editing (equal). **Koichi Fukunaga:** Funding acquisition (equal); project administration (equal); writing – original draft (equal); writing – review and editing (equal).

## FUNDING INFORMATION

This study was supported by the Japan Society for the Promotion of Science to H.T. 22K08289 and the MSD Life Science Foundation, Public Interest Incorporated Foundation. This study was also partly supported by the National Research Foundation of Korea (NRF) grant funded by the Korean government (MSIT) [grant number 2022R1A2C1092956].

## CONFLICT OF INTEREST STATEMENT

The authors have no conflict of interest.

## ETHICS STATEMENT

This research protocol was approved by the Institutional Review Board of Keio University Hospital (approval numbers: 2022‐1028 and 2011‐0171) and the Institutional Review Board of Yeouido St. Mary Hospital (approval number: SC18RNSI0037).

## PATIENT CONSENT

We collected clinical information from eligible individuals with the opportunity for patients to refuse to participate in this study (opt‐out method). Individual informed consent was obtained from those who underwent detailed analysis with the original sequence data of ODxTT.

## CLINICAL TRIAL REGISTRATION

The study protocol is registered at the UMIN clinical trials registry (UMIN000050424).

## Supporting information


Figure S1.

Figure S2.

Figure S3.

Table S1.

## Data Availability

The data that support the findings of this study are available on request from the corresponding author. The data are not publicly available due to privacy or ethical restrictions.
