# Supplementary material for: Clinical utility of the Oncomine Dx Target Test multi‐CDx system and the possibility of utilizing those original sequence data
Source: Cancer Med. 2024 Mar 8;13(4):e7077. doi: 10.1002/cam4.7077 (PMC10922029; doi:10.1002/cam4.7077)
Supplement: Supplementary file 1 — Figure S1. [file CAM4-13-e7077-s004.pptx]

## Slide 1
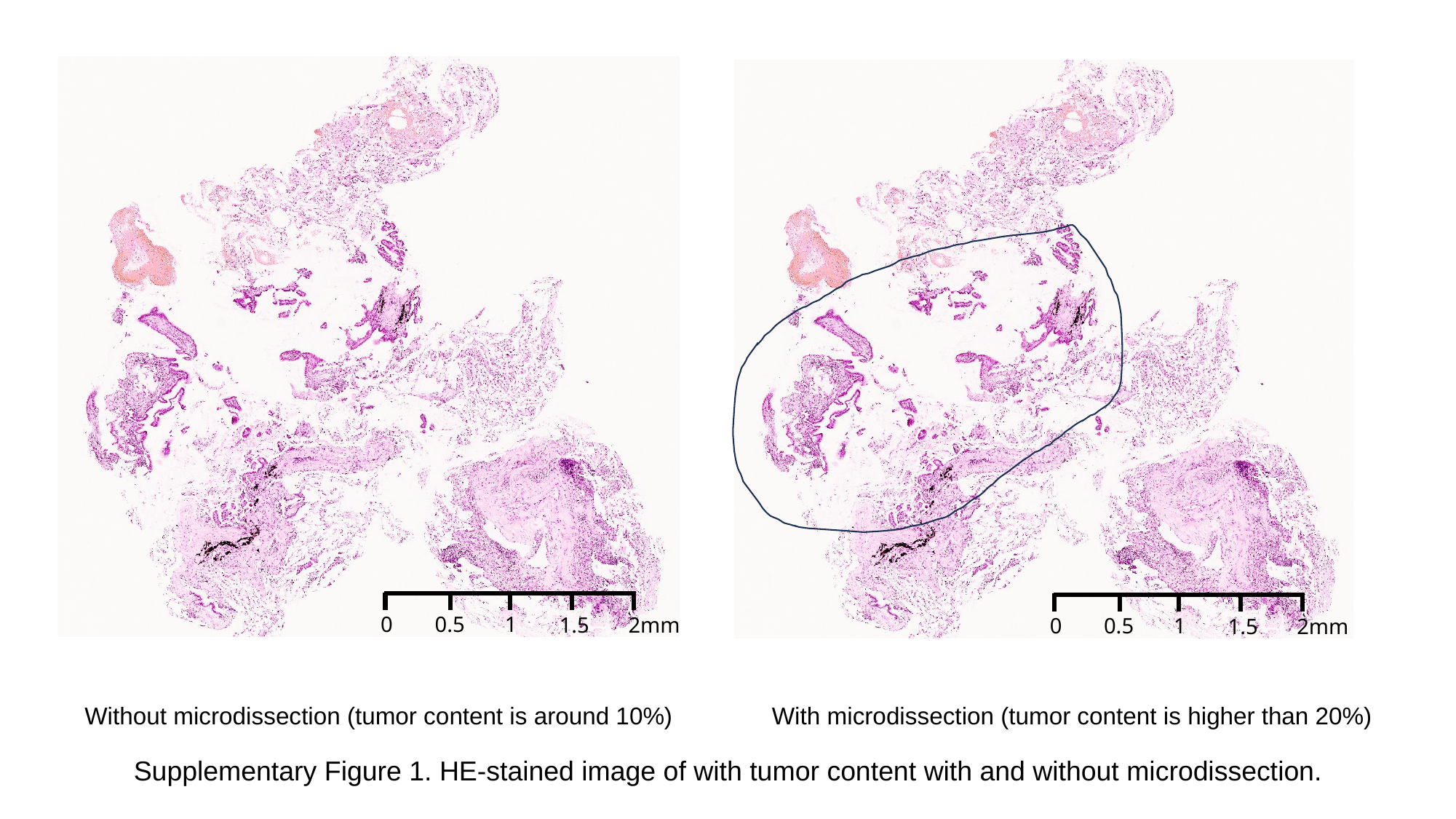

0
0.5
1
1.5
2mm
0
0.5
1
1.5
2mm
Without microdissection (tumor content is around 10%)
With microdissection (tumor content is higher than 20%)
Supplementary Figure 1. HE-stained image of with tumor content with and without microdissection.
