# Supplementary material for: Clinical utility of the Oncomine Dx Target Test multi‐CDx system and the possibility of utilizing those original sequence data
Source: Cancer Med. 2024 Mar 8;13(4):e7077. doi: 10.1002/cam4.7077 (PMC10922029; doi:10.1002/cam4.7077)
Supplement: Supplementary file 2 — Figure S2. [file CAM4-13-e7077-s001.pptx]

## Slide 1
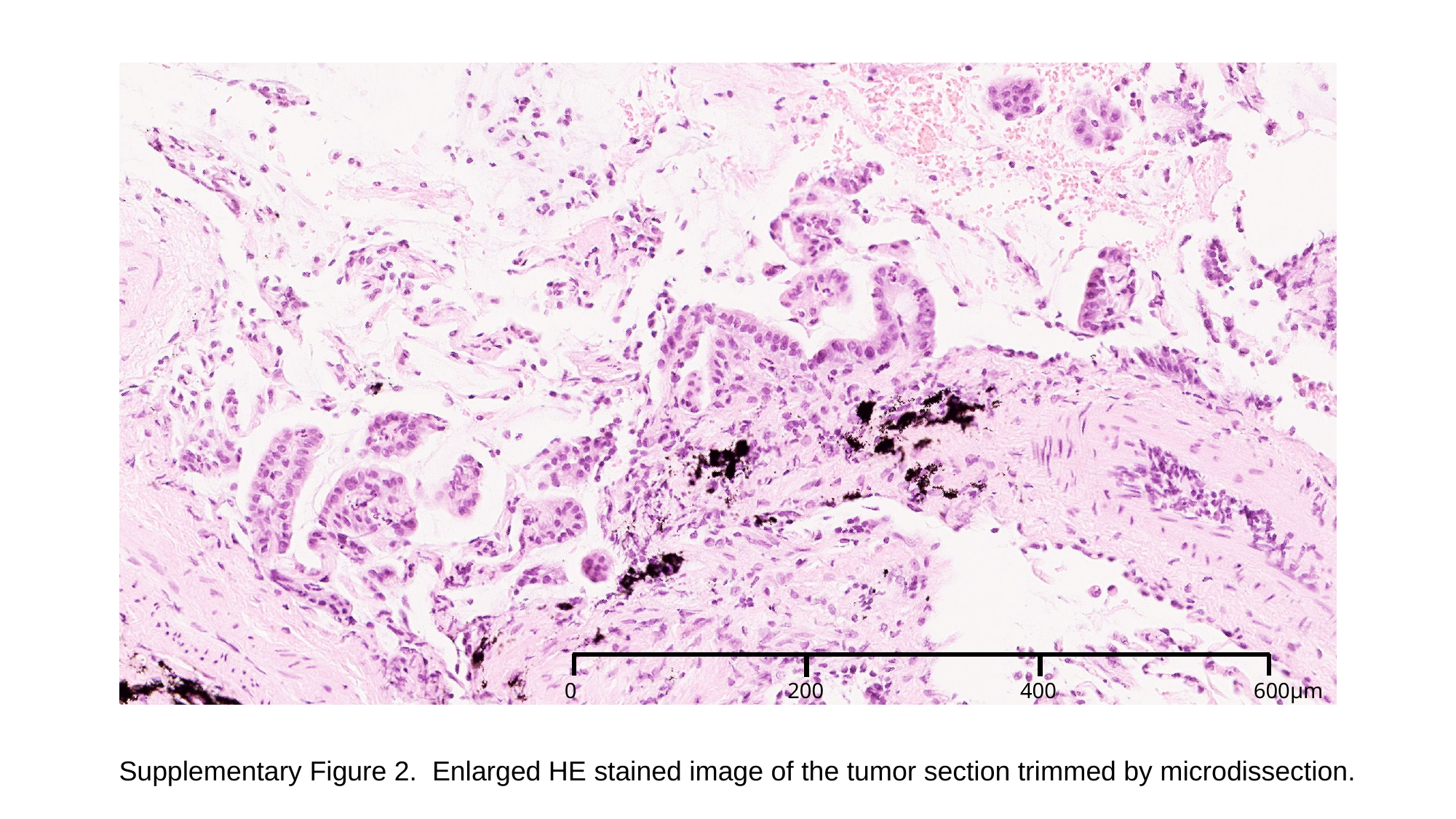

200
400
600μm
0
Supplementary Figure 2. Enlarged HE stained image of the tumor section trimmed by microdissection.
