# Supplementary material for: Clinical utility of the Oncomine Dx Target Test multi‐CDx system and the possibility of utilizing those original sequence data
Source: Cancer Med. 2024 Mar 8;13(4):e7077. doi: 10.1002/cam4.7077 (PMC10922029; doi:10.1002/cam4.7077)
Supplement: Supplementary file 3 — Figure S3. [file CAM4-13-e7077-s002.pptx]

## Slide 1
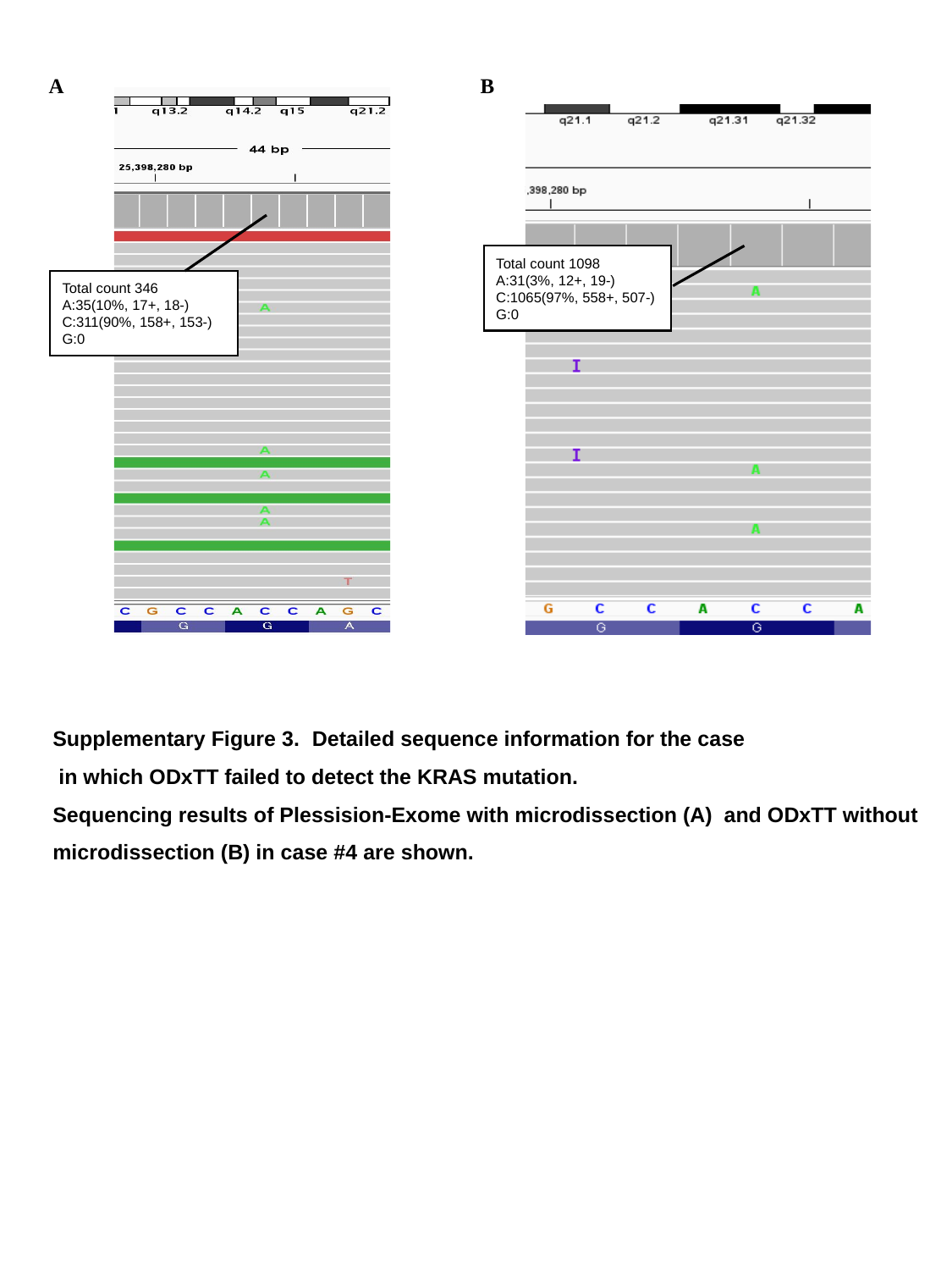

A
B
Total count 1098
A:31(3%, 12+, 19-)
C:1065(97%, 558+, 507-)
G:0
Total count 346
A:35(10%, 17+, 18-)
C:311(90%, 158+, 153-)
G:0
Supplementary Figure 3. Detailed sequence information for the case
 in which ODxTT failed to detect the KRAS mutation.
Sequencing results of Plessision-Exome with microdissection (A) and ODxTT without
microdissection (B) in case #4 are shown.
